# The Expansion of Myeloid-Derived Suppressor Cells Is Associated with Joint Inflammation in Rheumatic Patients with Arthritis

**DOI:** 10.1155/2018/5474828

**Published:** 2018-06-26

**Authors:** Junqing Zhu, Shixian Chen, Lisheng Wu, Ran Wang, Songyuan Zheng, Di Zhao, Xiangyang Wang, Juan Li

**Affiliations:** ^1^Department of Rheumatology, Nanfang Hospital, Southern Medical University, Guangzhou, Guangdong 510515, China; ^2^Department of Internal Medicine of Traditional Chinese Medicine, College of Traditional Chinese Medicine, Southern Medical University, Guangzhou, Guangdong 510515, China; ^3^Department of Human and Molecular Genetics, Virginia Commonwealth University School of Medicine, Richmond, VA 23220, USA

## Abstract

**Introduction:**

We investigated the proportion of myeloid-derived suppressor cells (MDSCs) and their subsets in patients with rheumatic diseases and clarified the association between these cells and the patient clinical data.

**Methods:**

Patients with rheumatic diseases and healthy controls were recruited. The clinical characteristics were obtained. The MDSCs and their subsets were marked with fluorescently labelled antibodies and were then analyzed with flow cytometry.

**Results:**

The patients included 31 with RA, 21 with AS, 14 with OA, 11 with SLE with arthritis, 13 with SLE without arthritis, 9 with Gout, 10 with HUA, and 25 healthy controls. The proportions of MDSCs, M-MDSCs, and G-MDSCs were higher in patients with RA than in healthy controls (6.56±6.77% versus 1.46±0.96%, 2.52±3.81% versus 0.35±0.35%, and 1.13±1.64% versus 0.18±0.14%; p<0.001). The same increased cells were also found in other patients. The proportions of MDSCs and M-MDSCs were mostly correlated with the patient's joint inflammation indexes and the disease activity. When other cell subsets were adjusted, the increased risk of arthritis was also obtained for M-MDSCs (adjusted OR=5.772; p=0.031).

**Conclusions:**

The expansion of MDSCs and their subsets was correlated with the disease activity and joint inflammation in patient with different rheumatic diseases. The proportion of M-MDSCs was associated with the risk of arthritis in those populations.

## 1. Introduction

Myeloid-derived suppressor cells (MDSCs) are a heterogeneous population of immature myeloid cells with a remarkable ability to suppress the immune system [1]. These cells were first observed in the microenvironment of tumor-bearing mice and were found to play an important role in tumor growth by suppressing the antitumor immune responses [2]. Murine MDSCs are characterized by the expression of CD11b and Gr-1 on the cell surface and are further divided into two subsets including a monocytic MDSC (M-MDSC) with the CD11b^+^LY6G^−^LY6C^high/+^ phenotype and a granulocytic MDSC (G-MDSC) with the CD11b^+^LY6G^+^LY6C^low/−^ phenotype [3, 4]. In humans, MDSCs are most commonly marked as CD11b^+^CD33^+^HLA^−^DR^low/−^ cells, while CD14 and CD15 have been further suggested to be markers for M-MDSCs and G-MDSCs, respectively [5, 6]. In spite of the identification of various MDSC markers and their subsets, the cells are heterogeneous and display different functions depending on the actual pathological conditions [7].

In practice, MDSCs have become a research hotspot because of their expansion and function under different pathological conditions in cancer [2], as well as infection [8, 9], chemotherapy [10], and autoimmune disease [11]. Recent studies have revealed that the expansion of circulating MDSCs and their subsets is correlated with the systemic lupus erythematosus (SLE) disease activity index (SLEDAI) scores in patients with SLE [12, 13]. In addition, higher levels of MDSC-like cells have been detected in patients with inflammatory bowel disease (IBD) and appear to correspond with the disease activity [14]. Although the MDSC expansion has been demonstrated in the peripheral blood and synovial fluid of patients with rheumatoid arthritis (RA) [4], the role of MDSCs in collagen-induced arthritis (CIA) mouse models remains controversial. Our previous study found that MDSCs from a CIA mouse model have the capacity to stimulate Th17 cell differentiation and lead to the progression of arthritis [15]. Other studies in a mouse RA model revealed that MDSCs exert their suppressive functions by inhibiting the proliferation of CD4^+^ T cells and that the adoptive transfer of MDSCs can decrease the severity of CIA [16, 17]. In brief, although MDSCs were found to suppress the T cell response of some cancer patients and tumor-bearing mice, the role of those cells in rheumatic diseases is less well-understood. Nevertheless, most rheumatic diseases have a common pathological process, such as inflammatory arthritis and systemic tissue damage [18]. Whether or not MDSCs and their subsets are abnormally expressed in patients with inflammatory-associated rheumatic diseases is still unclear.

The purpose of our study was not only to investigate the proportion of MDSCs and their subsets in patients with different rheumatic diseases, but also to clarify the correlation between the numbers of these cells and the patient clinical data, including inflammatory markers, disease activity indexes, and other disease-related specificity indexes. Additionally, this study also explores the risk of arthritis associated with the proliferation of MDSCs and their subsets in patients with rheumatic diseases.

## 2. Methods

### 2.1. Ethics Statement

This study was conducted according to the principles of the Declaration of Helsinki and approved by the Institutional Medical Ethics Review Board of Nanfang Hospital. All demographic and clinical characteristics from patients with rheumatic diseases were obtained after written consent was received.

### 2.2. Patients and Their Characteristics

One hundred and nine patients with rheumatic diseases and twenty-five healthy controls were recruited from the clinics of rheumatology or physical examination at Nanfang Hospital in China between September 2015 and October 2017. The rheumatic diseases in patients included rheumatoid arthritis (RA), as determined by the American Rheumatism Association (ARA) 1987 revised classification criteria [19], ankylosing spondylitis (AS), determined by the 1984 modified New York classification criteria [20], osteoarthritis (OA), determined by the 1986 ARA classification criteria [21], systemic lupus erythematosus (SLE), determined by the 1997 American College of Rheumatology (ACR) revised classification criteria [22], Gout, determined by the 1977 ARA preliminary criteria [23], and hyperuricemia (HUA), determined by the 2017 Chinese multidisciplinary consensus on the diagnosis and treatment of hyperuricemia and its related diseases [24]. All participants suffering solely from one of these rheumatic diseases were selected.

The common clinical characteristics included gender, age, disease duration, erythrocyte sedimentation rate (ESR, mm/h), C-reactive protein level (CRP, mg/L), total joint pain, as assessed on a visual analogue scale (0-10 cm), and swollen joint counts (n). In addition, the special clinical indexes for each rheumatic disease were also obtained. For patients with RA, this included health assessment questionnaire (HAQ) [25], disease activity score in 28 joints (DAS28) based on CRP [26, 27], rheumatoid factor (RF, IU/ml), and anti-citrullinated protein antibody (ACPA, U/ml). For patients with AS, it included human leukocyte antigen B27 (HLA-B27) positivity, ankylosing spondylitis disease activity score (ASDAS) [28], and bath ankylosing spondylitis functional index (BASFI) [29]. For patients with SLE, it included systemic lupus erythematosus disease activity index (SLEDAI) [30], antinuclear antibody (ANA, U/ml), anti-double stranded DNA antibody (Anti-dsDNA, U/ml), complement C3 (C3, g/L), and the presence of joint involvement. For patients with Gout + HUA, it included serum uric acid (SUA, *μ*mol/L). The percentage of patients who continue to take related drugs were also collected including nonsteroidal anti-inflammatory drugs (NSAIDs) for more than 1 week, disease-modifying antirheumatic drugs (DMARDs) for more than 3 months, glucocorticoid drugs (GCs) for more than 2 weeks, and uric-acid-lowering drugs (UALs) for more than 2 weeks.

### 2.3. Antibodies and Reagents

The phycoerythrin cyanine 7- (PE-Cy7-) conjugated mouse IgG1 anti-human CD33 (clone WM-53) and PE-Cy7-conjugated mouse IgG1 isotype matched control antibodies were purchased from eBiosciences (San Diego, California, USA). The allophycocyanin- (APC-) conjugated mouse IgG1 anti-human CD11b (clone ICRF44), V450-conjugated mouse IgG2a anti-human HLA-DR (clone G46-6), fluorescein isothiocyanate- (FITC-) conjugated mouse IgG2a anti-human CD14 (clone M5E2), phycoerythrin (PE)-conjugated mouse IgM anti-human CD15 (clone H198), and APC/V450/FITC/PE-conjugated mouse IgG1/IgG2a/IgM isotype matched control antibodies were purchased from BD (San Jose, California, USA). The 1-Step Fix/Lyse Solution (10×) was purchased from eBiosciences (San Diego, California, USA) and the phosphate-buffered saline (PBS) was purchased from GIBCO (Grand Island, New York, USA).

### 2.4. Flow Cytometric Analysis

As previously described [31], the whole blood was collected in EDTA (ethylenediaminetetraacetic acid) anticoagulant tubes and 10 *μ*l of each of the anti-CD11b/CD33/HLA-DR/CD14/CD15 fluorescently labelled antibodies was used to mark the cell surface molecules in 100 *μ*l of the whole blood for 30 min at 4°C. Isotype matched antibodies were used as the controls. The red blood cells were then lysed with 2 ml of room temperature 1-Step Fix/Lyse Solution (1×) for 20 min. Finally, the cells were resuspended in 300 *μ*l flow stain buffer. And the cell surface fluorescence intensity was analyzed on a FACSAria™ I (BD Bioscience, San Jose, California, USA).

### 2.5. Statistical Analysis

All statistics were calculated with SPSS (V.20, SPSS Inc., Chicago, USA) and the statistical charts were formulated with GraphPad Prism 5.0 (GraphPad Software Inc., San Diego, California, USA) and StataSE® v.12.0 (StataCorp, College Station, Texas, USA). Measurements data are presented as mean ± standard deviation (mean ± SD), while count data are presented as numbers (n) and the percentage (%). The Shapiro-Wilk test was used to check for normality and Levene's test was used to determine the homogeneity of variance in a small sample for the measurement data (3 ≤ n ≤ 50). A p<0.1 was considered statistically significant. Student's t-test was used to evaluate the statistical differences between groups when the distributions of data from both groups had equal variance, and the Welch-Satterthwaite approximate t-test was used when unequal variance was found. The nonnormal distribution measurement data were tested with the Wilcoxon rank-sum test. In order to count data, Pearson's Chi-square test was used for comparison and Fisher's exact test was used when theoretical frequency was less than 5 or the total observation frequency was less than 30. Pearson's and Spearman's Correlation analyses were performed to evaluate the associations between variables for the normal and nonnormal data, respectively. All correlation coefficients (r) and* p* values are reported. Bivariate analysis was performed for the unadjusted risk of arthritis from the frequency of MDSCs and their subsets. In addition, the binary logistic regression was used to identify the adjusted risk. The odds ratios (OR) and its 95% confidence interval (CI) have also been reported. A* p*<0.05 was considered statistically significant (*∗* or ^#^* p*<0.05, *∗∗* or ^##^* p*<0.01, and *∗∗∗* or ^###^* p*<0.001) and all p values were two tailed.

## 3. Results

### 3.1. Patient Characteristics

The characteristics of the included patients with rheumatic diseases and of the healthy controls are shown in Table 1. There were 31 RA, 21 AS, 14 OA, and 11 SLE with arthritis, 13 SLE without arthritis, 9 Gout, 10 HUA, and 25 healthy controls. Except for the gender of the SLE patients and the age of the OA patients, there were no statistically significant differences in gender or age for any of the groups when compared with the healthy controls. The average disease duration, ESR, CRP, total joint pain, and the swollen joint counts were also calculated for certain group of patients with rheumatic disease. Except for the CRP, the above clinical indexes (including disease duration and ESR) showed no statistical difference between the SLE groups with and without arthritis or between the Gout and HUA groups.

For patients with RA, the average HAQ scores, DAS28, IgM RF, and ACPA were 1.6±1.5, 3.7±1.0, 566.3±1027.9 IU/ml, and 58.6±38.0 U/ml, respectively. About seventy-six percent of AS patients were HLA-B27 positive. The average scores for these patients were 2.9±0.9 and 4.3±1.6 based on the ASDAS and BASFI assessments. In the SLE patients, the average SLEDAI, ANA, anti-dsDNA, and C3 were 6.6±3.7, 205.3±145.5 U/ml, 78.3±61.4 U/ml, and 0.6±0.3 g/L, respectively. No statistically significant differences were found for the clinical indexes between SLE patients with and without arthritis. The average SUA was 538.6±133.6 *μ*mol/L in pooled patients with Gout and HUA and no statistically significant differences were found between the two groups. At baseline, the number and percentage of patients who continue to take related drugs were also showed in Table 1.

### 3.2. The Expansion of MDSCs and Their Subsets in Patients with Rheumatic Diseases

**Figure 1:**
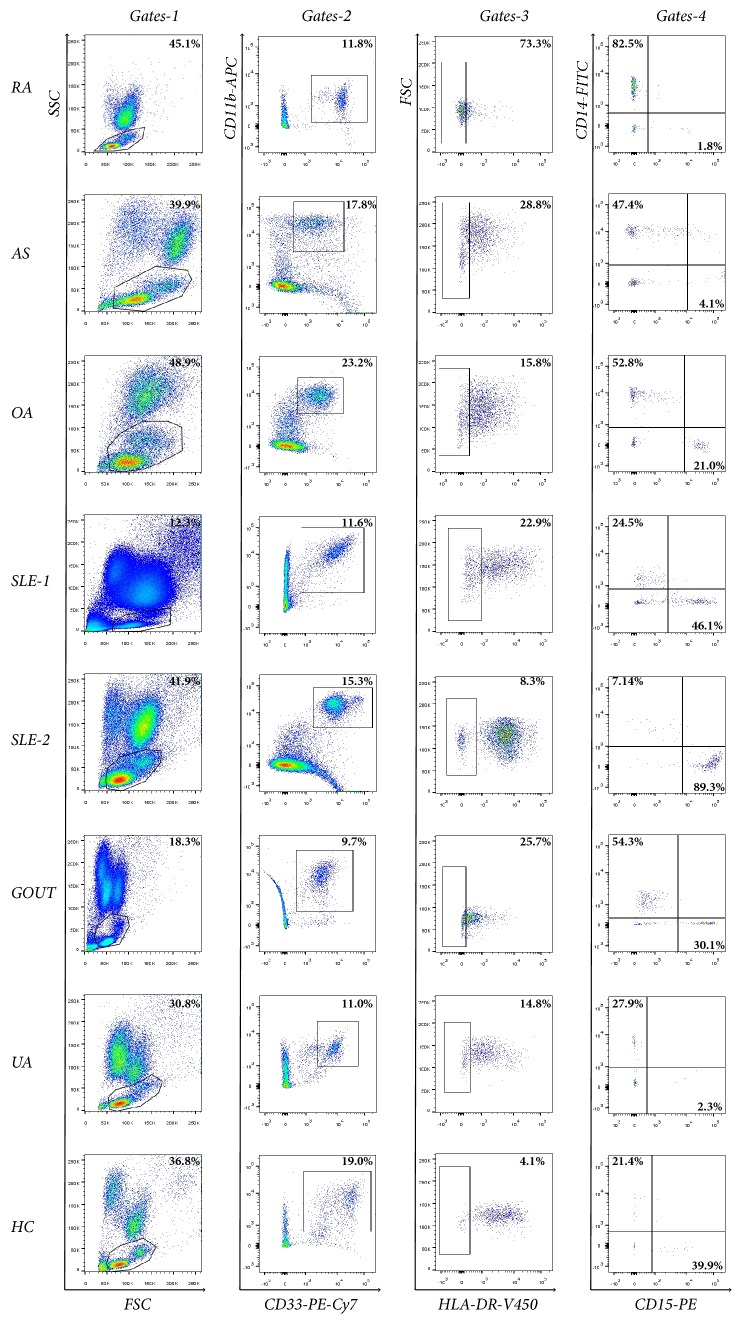
**The representative flow charts of myeloid-derived suppressor cells (MDSCs) and their subsets in the peripheral blood (PB) of rheumatic patients**. Cells were prepared from the PB of rheumatoid arthritis (RA), ankylosing spondylitis (AS), osteoarthritis (OA), systemic lupus erythematosus (SLE) with or without arthritis (SLE-1, SLE-2), Gout, and hyperuricemia (HUA) patients, as well as healthy controls (HC). They were then stained for CD11b, CD33, HLA-DR, CD14, and CD15. Lymphocytes and monocytes were defined by the forward and side scatter gates (Gates-1). Myeloid-derived suppressor cells (MDSCs) were defined by CD11b+CD33+HLA-DRlow/- (Gates-2, Gates-3). The proportions of monocytic MDSCs (M-MDSCs) and granulocytic MDSC (G-MDSCs) marked with CD14+ and CD15+ MDSCs, respectively, were penned in Gates-4.

**Figure 2:**
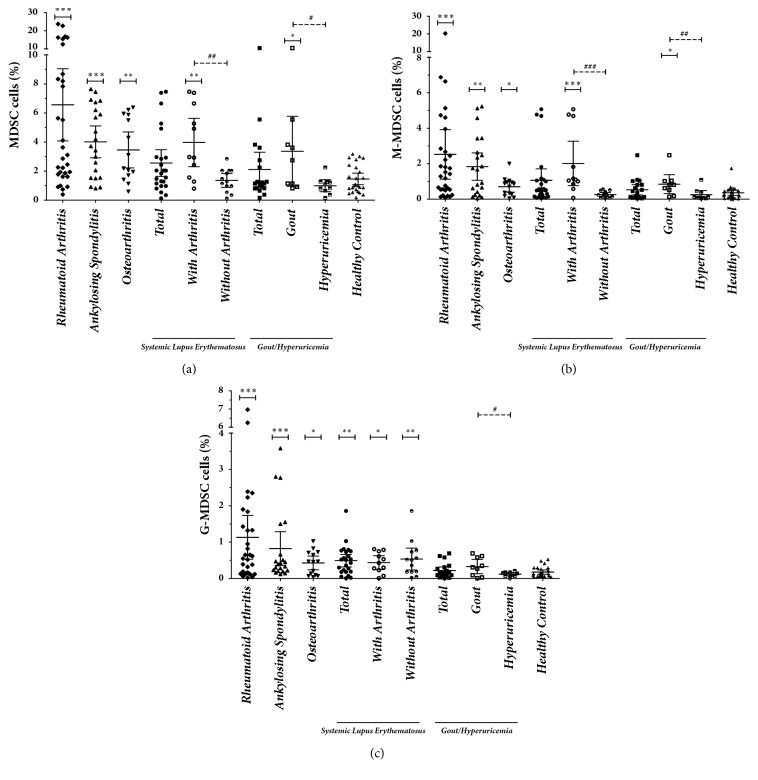
**The expansion of myeloid-derived suppressor cells (MDSCs) and their subsets in patients with rheumatic diseases**. The proportions of myeloid-derived suppressor cells (MDSCs) (a), monocytic MDSCs (M-MDSCs) (b), and granulocytic MDSC (G-MDSCs) (c) in patients with rheumatic diseases are shown in the scatter diagram. *∗* compared with healthy control; # compared with the systemic lupus erythematosus group with arthritis or Gout; *∗* or ^#^ p<0.05, *∗∗* or ^##^ p<0.01, and *∗∗∗* or ^###^ p<0.001.

The MDSC, M-MDSC, and G-MDSC phenotypes in the PB were analyzed using five color flow cytometry in patients with different rheumatic diseases, as well as in healthy controls (Figure 1). The proportions of the cells in different groups are shown in Table 1 and Figure 2. In the PB lymphocytes and monocytes, the proportion of MDSCs, M-MDSCs, and G-MDSCs was higher in patients with RA than in healthy controls (6.56±6.77% versus 1.46±0.96%, 2.52±3.81% versus 0.35±0.35%, and 1.13±1.64% versus 0.18±0.14%; p<0.001). The same increase of MDSCs and their subsets was also found in patients with AS and OA when compared with healthy controls (p<0.05). However, no significant statistical differences were found in the proportion of MDSCs and their subsets between healthy controls and patients with SLE or Gout/HUA (p>0.05), except for the increased numbers of G-MDSCs in SLE patients (0.49±0.41% versus 0.18±0.14%; p=0.002).

Further, subgroup analysis was performed in patients with SLE or Gout/HUA according to the condition of joint involvement (Table 1 and Figure 2). The proportions of MDSCs, M-MDSCs, and G-MDSCs in SLE patients with arthritis were 3.97±2.47%, 2.01±1.87%, and 0.44±0.28%, which increased significantly when compared with healthy controls (p=0.002, p<0.001, p=0.012). The same differences were found between SLE patients with and without arthritis with respect to the proportion of MDSCs and M-MDSCs (3.97±2.47% versus 1.36±0.79%, 2.01±1.87% versus 0.26±0.18%; p=0.006, p<0.001), but not for the proportion of G-MDSCs (0.44±0.28% versus 0.54±0.50%; p=1.0). Similarly as in the group of total SLE patients, no significant statistical differences were found in the proportion of MDSCs and their subsets between healthy controls and SLE patients without arthritis, except for the increased proliferation of G-MDSCs. The proportions of MDSCs and M-MDSCs in patients with Gout were 3.36±3.13% and 0.84±0.70%, which was higher than found in both healthy controls (p=0.037, 0.012) and patients with HUA (p=0.028, 0.006). The proportion of G-MDSCs in patients with Gout was also higher than in those with HUA (0.33±0.26% versus 0.12±0.06, p=0.044). No significant statistical differences were found in the proportion of MDSCs and their subsets between healthy controls and patients with HUA (p>0.05).

### 3.3. The Correlation of MDSCs and Their Subsets to Patient Clinical Variables

In order to clarify the correlation between the proportion of MDSCs and patient clinical variables, a correlation analysis was performed for each group of patients with different rheumatic diseases (Table 2). The proportions of MDSCs and M-MDSCs were correlated with CRP in RA patients (r=0.379, 0.594; p=0.036, p<0.001), AS patients (r=0.494, 0.801; p=0.023, p<0.001), OA patients (r=0.877, 0.746; p<0.001, p=0.002), and Gout patients (r=0.762, 0.883; p=0.017, p=0.002). Nevertheless, no significant correlation was found between those two cell subsets and CRP in SLE patients without arthritis or in HUA patients. Those two cell subsets were also correlated with total joint pain in RA (r=0.529, 0.766; p=0.002, p<0.001), AS (r=0.707, 0.799; p<0.001), OA (r=0.813, 0.844; p<0.001), SLE with arthritis (r=0.761, 0.934; p=0.007, p<0.001), and Gout patients (r=0.795, 0.911; p=0.01, p=0.001). The proportions of M-MDSCs were correlated with swollen joint counts in RA patients (r=0.371; p=0.040), OA patients (r=0.590; p=0.026), SLE patients with arthritis (r=0.918; p<0.001), and Gout patients (r=0.979, p=0.002).

In addition, the special clinical indexes for each rheumatic disease were also analyzed with their correlation to MDSCs and MDSC subsets (Table 2). For patients with RA, the proportions of MDSCs and M-MDSCs were correlated with DAS28 (r=0.481, 0.749; p=0.006, p<0.001) and ACPA (r=0.475, 0.668; p=0.007, p<0.001). Similarly, the proportions of MDSCs and M-MDSCs were correlated with ASDAS in patients with AS (r=0.596, 0.908; p=0.004, p<0.001). Other correlation analysis results between MDSCs and their subsets and patient clinical variables were found in groups of patients with different rheumatic diseases Table 2.

### 3.4. The Association of Arthritis Risk with the Proportion of MDSCs and Their Subsets in Rheumatic Patients

**Figure 3:**
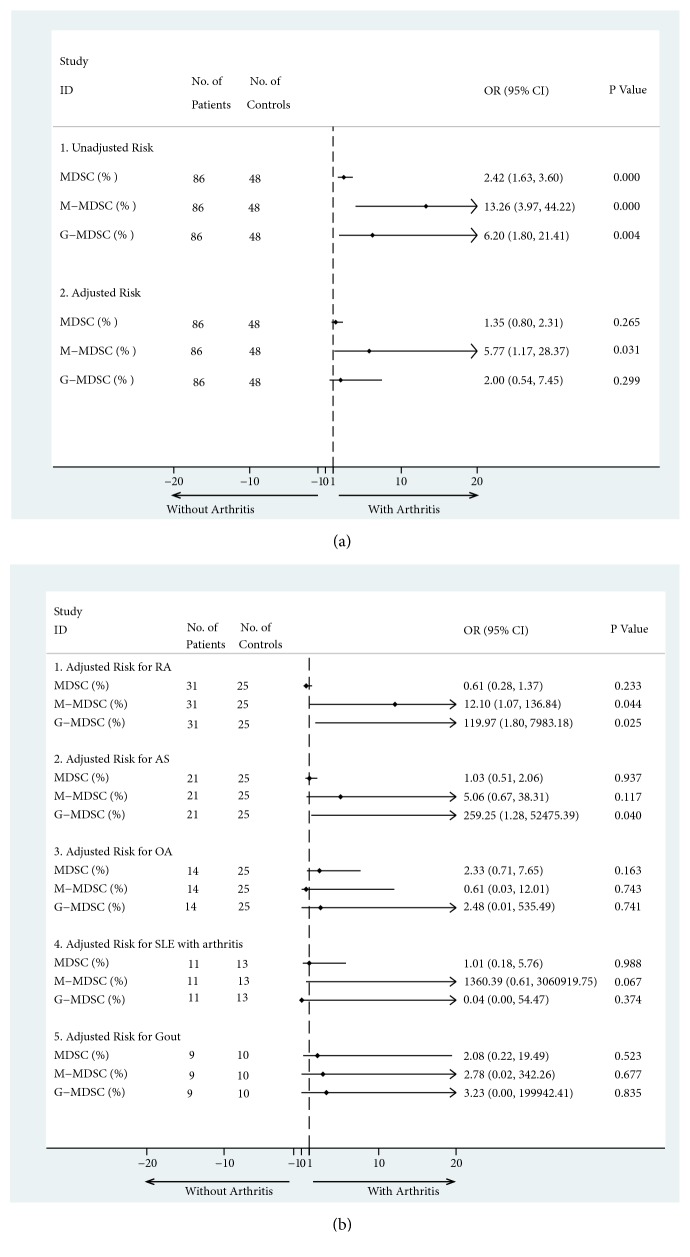
**The association between the risk of arthritis and the expansion of myeloid-derived suppressor cells (MDSCs), as well as their subsets**. (a) The pooled risk of arthritis from myeloid-derived suppressor cells (MDSCs) and their subsets. There were 86 patients in arthritis group, including RA, AS, OA, SLE with arthritis, and Gout patients. There were 48 controls, including SLE patients without arthritis, HUA patients, and healthy controls. Both bivariate analysis (a-1) and the binary logistic regression (a-2) were performed. (b) Subgroups analysis with the binary logistic regression for the risk of arthritis was grouped by the different arthritis (b). MDSCs, myeloid-derived suppressor cells; M-MDSCs, monocytic MDSCs; G-MDSCs, granulocytic MDSCs; RA, rheumatoid arthritis; AS, ankylosing spondylitis; OA, osteoarthritis, SLE, systemic lupus erythematosus; OR, odds ratios; 95% CI, 95% confidence interval.

In order to evaluate the risk of arthritis from the MDSCs and their subsets, all patients and healthy controls in this study were divided into two groups. One group with arthritis was comprised of 86 individuals, including RA, AS, OA, SLE with arthritis, and Gout patients, while the other group without arthritis was comprised of 48 individuals including SLE patients without arthritis, HUA patients, and the healthy controls. In the bivariate analysis, the frequency of MDSCs and their subsets were associated with the risk of arthritis (Figure 3(a)). The unadjusted OR was 2.417 (95% CI 1.625-3.597; p<0.001) for MDSCs, 13.257 (95% CI 3.974-44.223; p<0.001) for M-MDSCs, and 6.204 (95% CI 1.798-21.406; p=0.004) for G-MDSCs. The binary logistic regression was also used to identify the effect of potential risk factors from each cell subset adjusted for others (Figure 3(a)). Our results showed that the proportion of M-MDSCs was a risk factor when other cell subsets were adjusted (adjusted OR=5.772; 95% CI 1.174-28.369; p=0.031). However, the proportion of MDSCs and G-MDSCs did not serve as risk factors (adjusted OR=1.353, 2.004; 95% CI 0.795-2.305, 0.539-7.445; p=0.265, 0.299) for arthritis after adjustment. Further, the binary logistic regressions were performed in subgroups with different arthritis. The adjusted risks were also found for M-MDSCs and G-MDSCs in RA (adjusted OR=12.104, 119.97; 95% CI 1.071-136.839, 1.803-7983.18; p=0.044, 0.025), although the 95% CI is too wide due to the limitations of sample size and confounding factors (Figure 3(b)).

## 4. Discussion

### 4.1. The Cell Phenotype of MDSCs and Their Subsets

MDSCs were first studied in cancers and have typically been described as heterogeneous immature myeloid cells with immunosuppressive properties [2]. Under pathological conditions, such as infection [8, 9], chemotherapy [10], and autoimmune disease [11], MDSCs have been shown to play an important role in the occurrence and development of the disease. Due to the variety of pathological conditions, there is great disunity in the phenotypes and functions of MDSCs, especially in humans. For example, MDSCs identified by CD11b^+^CD33^low^HLA-DR^−^CD3^−^ in patients with bladder cancer have been correlated with clinical grade, stage, and poor prognosis [32]. In patients with acute-on-chronic liver failure, human CD14^+^CD15^−^HLA-DR^−^ MDSCs impair antimicrobial responses [33]. Even in the same disease models, such as in collagen-induced arthritis (CIA) in DBA/1J mice, the ratios of CD11b^+^Gr-1^high^ MDSCs and CD11b^+^Gr-1^medium^ MDSCs varied during the development of arthritis [34]. Nevertheless, an initial framework for the characterization of MDSCs was defined as cells expressing both CD11b and Gr-1 (including Ly6C and Ly6G) markers in mice [7]. Similarly, human MDSCs marked as CD33^+^HLA-DR^low/−^ are believed to contain more immature progenitors [35]. Other M-MDSCs are defined as CD11b^+^HLA^−^DR^low/−^CD14^+^CD15^−^ and G-MDSCs as CD11b^+^CD14^−^CD15^+^ [35]. Therefore, we use the term MDSCs to define cells marked with CD11b^+^CD33^+^HLA-DR^low/−^ and define M-MDSCs as CD11b^+^CD33^+^HLA-DR^−/low^CD14^+^CD15^−^ and G-MDSCs as CD11b^+^ CD33^+^ HLA-DR^−/low^CD15^+^ CD14^−^ in the investigation of the proliferation of these cells in different rheumatic diseases and their correlation with patient clinical data.

### 4.2. The Increased MDSCs and Their Subsets in Autoimmune Diseases

Previous studies have revealed that the frequency of MDSCs and their subsets increased in a variety of autoimmune diseases in mouse models, including models for type I autoimmune diabetes, multiple sclerosis, autoimmune hepatitis, IBD, SLE, and RA [4]. The proliferation of these cells is associated with disease activity or progression [4]. In both murine models of experimental autoimmune arthritis and in patients with RA, studies have determined that increased numbers of MDSCs are associated with the severity of joint inflammation [15, 34, 36, 37], while other studies found a negative correlation [16, 17]. In this study, the proportion of MDSCs and their subsets in PB lymphocytes and monocytes was higher in patients with RA than in healthy controls. The expansion of MDSCs and M-MDSCs, but not G-MDSCs, was correlated with disease activity and joint inflammation. The same expansion of MDSCs and M-MDSCs was also found in patients with AS and OA, which was correlated with joint inflammation indexes, such as CRP and total joint pain. Although, no abnormal increase in MDSCs or M-MDSCs was found in patients with SLE and Gout + HUA, similar results were obtained when the subgroup analysis was performed according to the condition of joint involvement. These findings indicate that MDSCs and their subsets may be associated with joint inflammation. Additionally, previous studies have revealed that the expansion of circulating MDSCs and their subsets is correlated with the SLEDAI scores in patients with SLE [12, 13]. However, our data showed that the proportion of MDSCs did not correlate with SLEDAI scores in SLE patient. And also the relationship between those cells and other symptoms in patients with SLE (such as skin lesions, serositis, and nephritis) is not clear. According to the above results, the role of MDSCs in SLE remains controversial.

### 4.3. The Increased Risk of Arthritis from MDSCs and Their Subsets

Further risk analysis revealed that the proportions of MDSCs and M-MDSCs were risk factors for arthritis in patients with rheumatic diseases. When other cell subsets were adjusted, the same risk was obtained for the expansion of M-MDSCs. This phenomenon may be explained by the common mechanism for the pathogenesis of arthritis. On the one hand, imbalances in the numbers and functions of CD4 T lymphocytes subsets (T helper 17 cells and regulatory T cells) are key pathogenic derangements in systemic rheumatic diseases [38]. A good deal of recent research has confirmed that the increased proliferation of MDSCs promotes the differentiation of T helper 17 cells and contributes to the progression of disease in both SLE [12, 13] and RA [15, 17, 34, 36]. On the other hand, although the exact mechanism of arthritis varies in different rheumatic diseases, a large amount of common cytokines, including tumor necrosis factor alpha (TNF*α*), interleukin-1*β* (IL-1*β*), IL-6, IL-17, and matrix metalloproteinase 3 (MMP3), mediates the process of joint inflammation [39–42]. This is consistent with recent studies of MDSCs in rheumatic diseases. For example, human MDSCs have been confirmed to be significantly increased in the synovial fluids of RA patients and to positively correlate with the levels of IL-17A [15]. MDSCs play a significant proinflammatory role in the pathogenesis of CIA by promoting Th17 cell differentiation from naïve CD4^+^ T cells in an IL-1beta-dependent manner [34]. Zhang H et al. have reported that MDSCs contributed to bone erosion by differentiating to osteoclasts in a RA mice model [43]. Taken together, these observations and our findings in this study suggest that MDSCs and their subsets play important roles in the development of arthritis via their interaction with cytokines or other immune cells.

### 4.4. The Limitations of This Study

There are several limitations to this study. First, although our studies have found positive correlations between the increased proliferation of MDSCs and their subsets with joint inflammation, patients with other types of arthritis (such as reactive arthritis and arthritis with inflammatory bowel disease) were not recruited. Second, due to the limitations of the number of patients with each rheumatic disease, the 95% CI for the risk of arthritis is too wide in subgroup analysis and the analysis of risk factors for arthritis was not adjusted by other clinical indexes. Larger sample size study and more correlation research (between those cells and cytokines or other immune cells) are needed. Third, this was the preliminary and observational study of correlation between MDSCs and arthritis. Cautious should be made for those results. Fourth, further studies are necessary in order to clarify the immunological role of MDSCs and their subsets in patients with different pathological conditions.

## 5. Conclusion

In conclusion, the present study clarifies the expansion of MDSCs and their subsets in different rheumatic patients, especially in those with arthritis. The proportion of those cells is correlated with patient disease activity and joint inflammation. Further analysis subsequently revealed that the proportions of MDSCs and M-MDSCs were risk factors for arthritis in the group with pooled patients. When other cell subsets were adjusted, the same risk was obtained for the increased proliferation of M-MDSCs. After subgroups analysis was divided by different disease, the adjusted risks were also found for M-MDSCs and G-MDSCs in RA.

## Figures and Tables

**Table 1:** Characteristics of the patients and controls included in the flow cytometric analysis.

Characteristics	Rheumatoid Arthritis	Ankylosing Spondylitis	Osteoarthritis	Systemic Lupus Erythematosus			Gout/Hyperuricemia			Healthy Control
				Total	With Arthritis	Without Arthritis	Total	Gout	Hyperuricemia	
Number of cases (n)	31	21	14	24	11	13	19	9	10	25
Gender (M/F) (n)	9/22	13/8	6/8	3/21*∗∗*	1/10*∗*	2/11*∗*	13/6	6/3	7/3	13/12
Age (years)	43.1±16.3	30.9±11.0	60.9±10.7*∗∗∗*	29.6±9.0	27.9±6.7	31.1±10.6	38.9±12.4	39.7±12.7	38.2±12.8	36.7±14.0
Disease Duration (years)	12.5±11.7	6.0±5.4	7.9±7.4	4.9±6.4	3.2±3.6	6.4±7.9	8.9±9.9	10.9±9.1	7.1±10.7	NA
ESR (mm/h)	31.7±30.5	14.9±11.8	19.6±9.6	25.1±18.6	25.3±19.4	25.0±18.6	10.9±5.9	12.7±7.9	9.4±2.8	NA
CRP (mg/L)	24.0±45.9	11.2±8.2	15.1±9.8	14.9±12.7	23.0±13.8	7.9±5.9^##^	9.1±7.6	13.7±8.6	4.8±2.4^###^	NA
Total Joint Pain (scores)	5.0±2.7	5.4±2.5	5.0±2.4	NA	6.6±2.7	NA	NA	4.4±1.5	NA	NA
Swollen Joint Counts (n)	1.6±1.5	NA	0.7±0.8	NA	2.0±1.7	NA	NA	1.4±0.9	NA	NA
HAQ (scores)	0.9±0.4	NA	NA	NA	NA	NA	NA	NA	NA	NA
DAS28 (scores)	3.7±1.0	NA	NA	NA	NA	NA	NA	NA	NA	NA
IgM RF (IU/ml)	566.3±1027.9	NA	NA	NA	NA	NA	NA	NA	NA	NA
ACPA (U/ml)	58.6±38.0	NA	NA	NA	NA	NA	NA	NA	NA	NA
HLA-B27 (+/-) (n)	NA	16/5	NA	NA	NA	NA	NA	NA	NA	NA
ASDAS (scores)	NA	2.9±0.9	NA	NA	NA	NA	NA	NA	NA	NA
BASFI (scores)	NA	4.3±1.6	NA	NA	NA	NA	NA	NA	NA	NA
SLEDAI (scores)	NA	NA	NA	6.6±3.7	5.5±2.5	7.5±4.4	NA	NA	NA	NA
ANA (U/ml)	NA	NA	NA	205.3±145.5	171.0±121.9	234.2±162.0	NA	NA	NA	NA
Anti-dsDNA (U/ml)	NA	NA	NA	78.3±61.4	71.1±54.5	84.1±68.3	NA	NA	NA	NA
C3 (g/L)	NA	NA	NA	0.6±0.3	0.6±0.2	0.6±0.1	NA	NA	NA	NA
SUA(*μ*mol/L)	NA	NA	NA	NA	NA	NA	538.6±133.6	507.1±126.1	566.9±140.4	NA
NSAIDs ^$^ (n, %)	20 (64.5%)	17 (81.0%)	8 (57.1%)	NA	6 (54.5%)	NA	NA	7 (77.8%)	NA	NA
DMARDs ^$^ (n, %)	24 (77.4%)	9 (42.9%)	NA	15 (62.5%)	7 (63.6%)	8 (61.5%)	NA	NA	NA	NA
GCs ^$^ (n, %)	NA	NA	NA	24 (100%)	11 (100%)	13 (100%)	NA	NA	NA	NA
UALs ^$^ (n, %)	NA	NA	NA	NA	NA	NA	16 (84.2%)	9 (100%)	7 (70%)	NA
MDSC (%)	6.56±6.77*∗∗∗*	4.04±2.41*∗∗∗*	3.46±2.14*∗∗*	2.56±2.18	3.97±2.47*∗∗*	1.36±0.79^##^	2.12±2.45	3.36±3.13*∗*	0.99±0.58^#^	1.46±0.96
M-MDSC (%)	2.52±3.81*∗∗∗*	1.84±1.70*∗∗*	0.70±0.53*∗*	1.06±1.53	2.01±1.87*∗∗∗*	0.26±0.18^###^	0.53±0.60	0.84±0.70*∗*	0.25±0.33^##^	0.35±0.35
G-MDSC (%)	1.13±1.64*∗∗∗*	0.82±1.02*∗∗∗*	0.43±0.33*∗*	0.49±0.41*∗∗*	0.44±0.28*∗*	0.54±0.50*∗∗*	0.22±0.21	0.33±0.26	0.12±0.06^#^	0.18±0.14

Note: ESR, erythrocyte sedimentation rate; CRP, C-reactive protein; HAQ, health assessment questionnaire; DAS28, disease activity score in 28 joints; RF, rheumatoid factor; ACPA, anti-citrullinated protein antibody; HLA-B27, human leukocyte antigen B27; ASDAS, ankylosing spondylitis disease activity score; BASFI, bath ankylosing spondylitis functional index; SLEDAI, systemic lupus erythematosus disease activity index; ANA, antinuclear antibody; Anti-dsDNA, anti-double stranded DNA antibody; C3, complement C3; SUA, serum uric acid; NSAIDs, nonsteroidal anti-inflammatory drugs; DMARDs, disease-modifying antirheumatic drugs; GCs, glucocorticoid drugs; UALs, uric-acid-lowering drugs; MDSC, myeloid derived suppressor cell; M-MDSC, monocytic MDSC; G-MDSC, granulocytic MDSC; Total Joint Pain was assessed on a visual analog scale (0-10 cm); NA, not available; ^$^ the percentage of patients treated with related drugs more than a specified period of time; ^**∗**^ compared with healthy control; ^#^ compared with the systemic lupus erythematosus group with arthritis or gout; *∗* or ^#^*p*<0.05, *∗∗* or ^##^*p*<0.01, and *∗∗∗* or ^###^*p*<0.001.

**Table 2:** The correlation between clinical variables in patients with rheumatic disease and the proportion of myeloid derived suppressor cells, as well as their subsets.

Correlation Analysis		MDSC		M-MDSC		G-MDSC	
		r	p	r	p	r	p
Rheumatoid Arthritis	Age (years)	-0.255	0.165	-0.239	0.195	-0.145	0.436
	Disease Duration (years)	0.007	0.972	0.052	0.781	-0.093	0.621
	ESR (mm/h)	0.265	0.150	0.322	0.077	0.081	0.665
	CRP (mg/L)	0.379	0.036*∗*	0.594	0.000*∗∗∗*	-0.095	0.611
	Total Joint Pain (scores)	0.529	0.002*∗∗*	0.766	0.000*∗∗∗*	0.142	0.446
	Swollen Joint Counts (n)	0.305	0.095	0.371	0.040*∗*	0.164	0.379
	HAQ (scores)	-0.003	0.987	0.160	0.390	0.066	0.722
	DAS28 (scores)	0.481	0.006*∗∗*	0.749	0.000*∗∗∗*	0.047	0.801
	IgM RF (IU/ml)	0.140	0.453	0.272	0.139	0.015	0.936
	ACPA (U/ml)	0.475	0.007*∗∗*	0.668	0.000*∗∗∗*	0.000	1.000

Ankylosing Spondylitis	Age (years)	-0.154	0.506	-0.211	0.359	-0.142	0.538
	Disease Duration (years)	-0.217	0.345	-0.214	0.352	-0.330	0.144
	ESR (mm/h)	0.087	0.709	-0.145	0.529	0.046	0.844
	CRP (mg/L)	0.494	0.023*∗*	0.801	0.000*∗∗∗*	0.043	0.854
	Total Joint Pain (scores)	0.707	0.000*∗∗∗*	0.799	0.000*∗∗∗*	-0.065	0.780
	ASDAS (scores)	0.596	0.004*∗∗*	0.908	0.000*∗∗∗*	0.042	0.858
	BASFI (scores)	0.228	0.320	0.251	0.273	-0.151	0.512

Osteoarthritis	Age (years)	0.051	0.863	0.055	0.852	-0.256	0.376
	Disease Duration (years)	0.073	0.805	-0.068	0.817	-0.276	0.34
	ESR (mm/h)	0.037	0.899	0.037$	0.900	-0.165$	0.573
	CRP (mg/L)	0.877	0.000*∗∗∗*	0.746$	0.002*∗∗*	0.408$	0.148
	Total Joint Pain (scores)	0.813	0.000*∗∗∗*	0.844	0.000*∗∗∗*	0.387	0.171
	Swollen Joint Counts (n)	0.330	0.250	0.590	0.026*∗*	0.186	0.525

Systemic Lupus Erythematosus With Arthritis	Age (years)	0.061$	0.858	-0.460	0.154	0.234$	0.489
	Disease Duration (years)	0.133	0.697	0.078	0.820	0.357	0.281
	ESR (mm/h)	0.173	0.611	0.378	0.252	-0.342	0.304
	CRP (mg/L)	0.555	0.077	0.855	0.001*∗∗*	-0.309	0.355
	Total Joint Pain (scores)	0.761$	0.007*∗∗*	0.934	0.000*∗∗∗*	0.101$	0.768
	Swollen Joint Counts (n)	0.551	0.079	0.918	0.000*∗∗∗*	-0.217	0.522
	SLEDAI (scores)	0.139$	0.683	0.161	0.636	0.607$	0.048*∗*
	ANA (U/ml)	-0.184$	0.589	0.000	1.000	0.335$	0.315
	Anti-dsDNA (U/ml)	0.203$	0.549	0.182	0.593	0.622$	0.041*∗*
	C3 (g/L)	0.229$	0.497	-0.328	0.352	-0.112$	0.743

Systemic Lupus Erythematosus Without Arthritis	Age (years)	0.059	0.847	-0.039	0.900	0.033	0.915
	Disease Duration (years)	-0.155	0.613	-0.077	0.801	0.109	0.722
	ESR (mm/h)	0.252	0.405	0.163	0.596	-0.072	0.816
	CRP (mg/L)	0.171	0.577	0.489	0.090	-0.149	0.628
	SLEDAI (scores)	0.038$	0.901	-0.196$	0.522	0.054	0.861
	ANA (U/ml)	0.219$	0.471	-0.067$	0.828	0.096	0.754
	Anti-dsDNA (U/ml)	-0.315$	0.295	-0.244$	0.421	-0.077	0.802
	C3 (g/L)	-0.433$	0.139	-0.580$	0.038*∗*	-0.432	0.141

Gout	Age (years)	-0.770	0.015*∗*	-0.683	0.042*∗*	-0.169$	0.665
	Disease Duration (years)	-0.218	0.574	-0.200	0.606	0.051$	0.897
	ESR (mm/h)	0.151	0.699	0.433	0.244	0.235$	0.543
	CRP (mg/L)	0.762	0.017*∗*	0.883	0.002*∗∗*	0.833	0.005*∗∗*
	Total Joint Pain (scores)	0.795	0.010*∗*	0.911	0.001*∗∗*	0.868$	0.002*∗∗*
	Swollen Joint Counts (n)	0.712	0.031*∗*	0.878	0.002*∗∗*	0.833	0.005*∗∗*
	SUA (*μ*mol/L)	-0.151	0.699	-0.183	0.637	0.082$	0.833

Hyperuricemia	Age (years)	0.077$	0.833	-0.123	0.736	0.330$	0.351
	Disease Duration (years)	0.091	0.803	0.018	0.960	0.049	0.894
	ESR (mm/h)	-0.395$	0.259	-0.326	0.358	-0.170$	0.640
	CRP (mg/L)	0.430	0.214	0.159	0.661	0.543	0.105
	SUA (*μ*mol/L)	-0.231$	0.522	-0.275	0.441	0.213$	0.554

Note: ESR, erythrocyte sedimentation rate; CRP, C-reactive protein; HAQ, health assessment questionnaire; DAS28, disease activity score in 28 joints; RF, rheumatoid factor; ACPA, anti-citrullinated protein antibody; ASDAS, ankylosing spondylitis disease activity score; BASFI, bath ankylosing spondylitis functional index; SLEDAI, systemic lupus erythematosus disease activity index; ANA, antinuclear antibody; Anti-dsDNA, anti-double stranded DNA antibody; C3, complement C3; SUA, serum uric acid; MDSC, myeloid derived suppressor cell; M-MDSC, monocytic MDSC; G-MDSC, granulocytic MDSC; Total Joint Pain was assessed on a visual analog scale (0-10 cm); r, all showed by Spearman's rank correlation coefficient, except when marked with $ (Pearson's correlation coefficient);. *∗p*<0.05, *∗∗p*<0.01, and *∗∗∗p*<0.001.

## Data Availability

The data used to support the findings of this study are available from the corresponding author upon request.
